# Pathogenic Adaptations Revealed by Comparative Genome Analyses of Two *Colletotrichum* spp., the Causal Agent of Anthracnose in Rubber Tree

**DOI:** 10.3389/fmicb.2020.01484

**Published:** 2020-07-21

**Authors:** Xianbao Liu, Boxun Li, Yang Yang, Jimiao Cai, Tao Shi, Xiaolan Zheng, Guixiu Huang

**Affiliations:** Environment and Plant Protection Institute, Chinese Academy of Tropical Agricultural Sciences (CATAS), Key Laboratory of Integrated Pest Management on Tropical Crops, Ministry of Agriculture, Key Laboratory for Monitoring and Control of Tropical Agricultural Pests, Haikou, China

**Keywords:** cell wall degrading enzymes, *Colletotrichum*, genome, *Hevea brasiliensis*, necrosis- and ethylene- inducing peptides, secondary metabolites associated genes, symptoms

## Abstract

*Colletotrichum siamense* and *Colletotrichum australisinense* cause *Colletotrichum* leaf disease that differ in their symptoms in rubber tree (*Hevea brasiliensis*), and pathogenicity of these two fungal species is also not identical on different cultivars of rubber tree. This divergence is often attributed to pathogen virulence factors, namely carbohydrate-active enzymes (CAZymes), secondary metabolites (SM), and small-secreted protein (SSP) effectors. The draft genome assembly and functional annotation of potential pathogenicity genes of both species obtained here provide an important and timely genomic resource for better understanding the biology and lifestyle of *Colletotrichum* spp. This should pave the way for designing more efficient disease control strategies in plantations of rubber tree. In this study, the genes associated with these categories were manually annotated in the genomes of *C. australisinense* GX1655 and *C. siamense* HBCG01. Comparative genomic analyses were performed to address the evolutionary relationships among these gene families in the two species. First, the size of genome assembly, number of predicted genes, and some of the functional categories differed significantly between the two congeners. Second, from the comparative genomic analyses, we identified some specific genes, certain higher abundance of gene families associated with CAZymes, CYP450, and SM in the genome of *C. siamense*, and Nep1-like proteins (NLP) in the genome of *C. australisinense.*

## Introduction

*Hevea brasiliensis* (Willd. ex A. Juss.) Müll. Arg., commonly known as the Para rubber tree, is native to the tropical rain forests of Central and South America, and the primary source of natural rubber. *Colletotrichum* leaf disease (CLD) caused by *Colletotrichum* species has become a serious biotic factor limiting rubber tree cultivation in plantations. The disease has been recorded and confirmed in most rubber growing countries such as China, Malaysia, Sri Lanka (Wastie, 1967; Liu et al., 1987; Jayasinghe et al., 1997). The fungus causing this disease had been originally identified as *Colletotrichum heveae* Petch, but then presumed to be *Colletotrichum gloeosporioides* (Penzig & Sacc.). Yet *C. gloeosporioides* as well as *Colletotrichum acutatum* are often associated with anthracnose disease occurrences in rubber trees (Brown and Soepena, 1994; Jayasinghe et al., 1997). Furthermore, *C. dematium*, *C. crassipes*, *C. karstii*, and *C. boninense* specimens have also been isolated from infected rubber leaves, but were deemed of less economic importance than the aforementioned two *Colletotrichum* species because they happen sporadically (Wastie and Janardhanan, 1970; Cai et al., 2016; Jiang et al., 2016).

Taxonomically, much of the recent research and treatments have primarily focused on studying the different *Colletotrichum* species complexes. According to the latest work and updates, the genus consists of fourteen species complexes (Jayawardena et al., 2016; Marin-Felix et al., 2017; Damm et al., 2018). Within just the last decade, many cryptic and new species have been reported from rubber leaves with CLD symptoms; for example, five *Colletotrichum* species belonging to the *C. acutatum* species complex were reported from Sri Lanka (Damm et al., 2012; Hunupolagama et al., 2017). In China, however, *Colletotrichum siamense*, *Colletotrichum fructicola*, and *Colletotrichum ledongense* of the *C. gloeosporioides* species complex were newly associated with CLD of rubber tree (Liu et al., 2018). Two new species (*Colletotrichum australisinense* and *C. bannaense*) of the *C. acutatum* species complex were also discovered, although *C. siamense* (the *C. gloeosporioides* species complex) and *C. australisinense* (the *C. acutatum* species complex) are currently recognized as the predominant species in that country. However, the infectious symptoms on rubber tree arising from *C. siamense* and *C. australisinense* are significantly different (Liu et al., 2018). The thorough characterization of species causing anthracnose and knowledge of their respective molecular mechanisms of infection is critical for devising strategies to effectively control the spread of the disease in rubber tree plantations.

The *Colletotrichum* species have evolved a diverse range of lifestyles to colonize and obtain nutrients from their host species of monocot and/or dicot plants, ranging functionally from biotrophs, nectrotrophs, hemibiotrophs to endophytes (De Silva et al., 2017). In recent years, progress has advanced toward a better understanding of their infection process at the molecular level and its correlation with the life history of each fungus (Villa-Rivera et al., 2017). The entire genomic sequence of some *Colletotrichum* species belonging to different species complexes, are now publicly available, providing an impetus to research of *Colletotrichum*–host tree interactions. Genus-wide comparative genome analyses of *Colletotrichum* species focusing on important gene classes, such as secretory proteases and carbohydrate-active enzymes (CAZymes), candidate effectors, and secondary metabolite (SM) biosynthesis genes, have revealed the core genes conserved in the genus *Colletotrichum*. Additionally, information on the amplification and contraction of certain gene families that could be attributed to their specific life histories and host range have been provided (O’Connell et al., 2012; Gan et al., 2013; Baroncelli et al., 2016; Gan et al., 2016; Buiate et al., 2017; Rao and Nandineni, 2017; Liang et al., 2018).

In this study, we report on the genome sequences of *C. siamense* in the *C. gloeosporioides* species complex (CGsc) and of *C. australisinense* in the *C. acutatum* species complex (CAsc), which, respectively, cause different symptoms in diseased rubber trees. Comparative genomic analyses were carried out between the two species. We laid special emphasis on analyzing the gene families encoding CAZymes, SM biosynthesis, and necrosis and ethylene-inducing peptide 1 (Nep1)-like proteins (NLPs), given their already known roles in plant–pathogen interactions, and characterized the content of these genes and their variation. Comparative genomics revealed reductions of gene families encoding CAZymes and SM biosynthesis within *C. australisinense* belonging to the CAsc. We also found evidence for an expansion of NLPs. Based on these patterns, we hypothesize that for *Colletotrichum* species with a broad host range, particularly those within CAsc, Lineage Specific Effector protein Candidates (LSECs) have diminished roles in interactions with plants, perhaps instead relying mainly on CAZymes, proteases, and NLPs for successful host colonization.

## Materials and Methods

### Fungal Cultures and Infection Conditions

*Colletotrichum australisinense* GX1655 and *C. siamense* HBCG01 isolates were obtained from distinctive leaf lesions associated with the different disease symptoms (the brown conical or raised spots vs. the anthracnose spots) they, respectively, cause in rubber trees. Their species identity was confirmed by a polyphasic approach (Liu et al., 2018). The GX1655 isolate was cultured on potato dextrose agar (PDA) and preserved as conidial suspension amended with 15% glycerol at –80°C. It was afterward deposited in the China General Microbiological Culture Collection center (CGMCC) under the accession number CGMCC3.18886.

### Genome Sequencing and Assembly

Genomic DNA of freshly collected mycelia was extracted with the QIAGEN^®^ Genomic DNA kit. The BluePippin system was used for the library construction. Genome sequencing was performed on a GridION X5 platform at the Nextomics Genomic Sequencing Center (Wuhan, China). Raw reads were trimmed with GraphMap and Minimap software tools^1^ to remove low quality reads and any reads having adaptor contamination (mean qscore >7). Clean reads were then *de novo* assembled using Canu v1.3 (Koren et al., 2017), for which the correction of each assembly was then assessed using Bwa 0.7.12-r1039 (Walker et al., 2014) and “Benchmarking Universal Single-Copy Orthologs” (BUSCO v3.0) (Simão et al., 2015; Seppey et al., 2019). The generated genomes were deposited at NCBI as BioProjects under accession PRJNA597657 and PRJNA597926.

### Gene Prediction

The transposable elements present in assembled genome were identified using a combination of *de novo* and homology-based approaches. Specifically, RepeatMasker v4.0.5 (Tarailo-Graovac and Chen, 2009) and RepeatProteinMask (v4.0.5, a package within RepeatMasker) were used to identify known sequences within the DNA repeat database (RepBase v21.01) (Bao et al., 2015). For the *de novo* predictions, RepeatModeler V1.0.8 and LTRfinder^2^ were jointly employed to build a *de novo* repeat library, after which RepeatMasker identified the repeats by using both the *de novo* repeat database and RepBase. Tandem repeats were annotated with RepeatMasker and the Tandem Repeats Finder (TRF; v4.07) (Benson, 1999).

Initial gene models were derived as statistically combined consensus models from both *de novo* and homology-based predictions. For *de novo* predictions, we used a Combined approach of Augustus (v3.2.1) (Stanke et al., 2008), GeneId (v1.4) (Parra et al., 2000), GenScan (Burge and Karlin, 1998), SNAP (Korf, 2004) and Glimmer (Majoros et al., 2004). For the homology-based predictions, the protein sequences from *Colletotrichum nymphaeae* and *Colletotrichum simmondsii* in the CAsc (Baroncelli et al., 2016), *C. fructicola* in the CGsc (Gan et al., 2013), and *Colletotrichum higginsianum* (Zampounis et al., 2016) were mapped onto the target genome, by using TblastN with an *E*-value cutoff of 1e-5. The ensuing aligned sequences as well as corresponding query proteins were then filtered and transferred into GeneWise (Birney, 2004) and GeMoMa (Keilwagen et al., 2018) to search for their accurately spliced alignments. Gene models predicted by the *de novo* and homology approaches were integrated using EvidenceModeler (EVM) (Haas et al., 2008), to generate a consensus gene set.

### Functional Annotation of Genes

The predicted genes were annotated using five well known protein databases: Cluster of Orthologous Group of proteins (COG), gene ontology (GO), Swiss-Prot (using an *E*-value threshold = 1e-05), Non-Redundant Protein Database (NR), and Kyoto Encyclopedia of Genes and Genomes (KEGG). Carbohydrate-utilizing enzymes were identified and classified based on a BLASTP search against Carbohydrate-Active Enzyme (CAZy) database^3^ with a cut-off *E*-value of 1e-5 (Liang et al., 2018). The cytochrome P450 genes were identified by retaining only the best hits found, i.e., P450s with >40% shared identity were assigned to the same family, through a BLASTP search (using an *E*-value threshold = 1e-5) against the cytochrome P450 database^4^ (Fischer et al., 2007; Liang et al., 2018). Potential SM clusters were identified using antiSMASH fungal version 5.0.0rc1 (Medema et al., 2011). The adenylation domain (A domain) of non-ribosomal peptide synthetases (NRPS) proteins and polyketide synthases (PKS)-NRPS hybrids, the keto-synthase domains (KS) (also referred to as N- and C-terminal docking domains) of PKS proteins and PKS-NRPS hybrids were separately used for the phylogenetic analyses (Buiate et al., 2017). Necrosis and ethylene-inducing peptide 1-like proteins (NLPs) genes were identified and classed based on a search for the conserved motif GHRHDWE in the predicted proteins and a TBlastN search against the current assembly of genomes, by using the six previously published NLP sequences belonging to three different types (i.e., type-1, type-2, and type-3) (Kleemann et al., 2012; Oome and Van den Ackerveken, 2014). Only full-length protein sequences were used in that analysis. Those proteins transported out of the cell and into the extracellular space were identified using a battery of tools: namely, Signal-P (v4.1) (Petersen et al., 2011), TargetP^5^ TMHMM (v2.0) (Krogh et al., 2001), GPI-SOM (Fankhauser and MaÈser, 2005), and WoLF PSORT (v0.2) (Horton et al., 2007), which were run sequentially.

### Construction of Phylogenetic Trees

For phylogenetic analysis, the genomes and/or proteomes of 10 *Colletotrichum* species (Gan et al., 2013; Baroncelli et al., 2014a, b, 2016; Hacquard et al., 2016; Zampounis et al., 2016) were downloaded from NCBI. Predicted proteins encoded by a total of 12 *Colletotrichum* species genomes were filtered, and clustered into orthologous groups by Orthofinder version 2.3.8 (Emms and Kelly, 2019). Single copy ortholog groups were then extracted for phylogenomic tree construction. Independent ortholog groups were aligned with MAFFT version 7^6^ and then concatenated (Baroncelli et al., 2016). A substitution model and its parameter values were selected using ProtTest version 3.4 (Abascal et al., 2005; Baroncelli et al., 2016). A maximum-likelihood (ML) phylogenetic tree with 1000 bootstraps was constructed with RAxML version 8.1.1 (Stamatakis, 2006; Liang et al., 2018). *Verticillium alfalfae* (Klosterman et al., 2011) was used as outgroup. In the phylogenetic analysis of SM clusters and NLP between *C. australisinense* and *C. siamense*, amino acid sequences were aligned using MUSCLE of MEGA 6, for which the best-fitting amino-acid substitution model and parameter settings were chosen based on the “model test” of MEGA 6. To construct each phylogenetic tree, the maximum likelihood algorithm implemented in MEGA 6 was used (Kumar et al., 2016), with *n* = 1000 bootstrap replications.

## Results

### Infection Conditions

**FIGURE 1:**
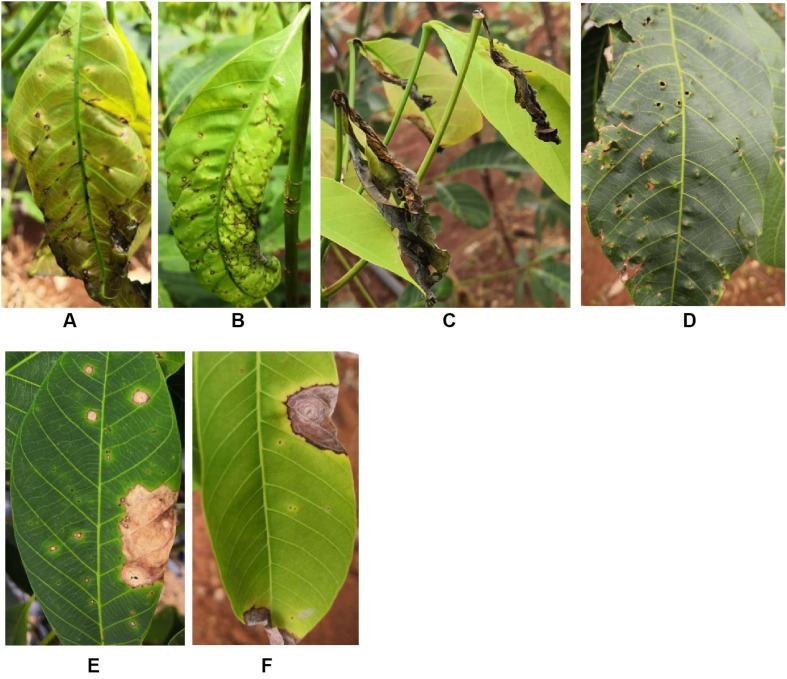
Symptoms caused by *Colletotrichum siamense* and *Colletotrichum australisinense* on rubber trees in the field. **(A,B,D)** Raised or wrinkled spots caused by *C. australisinense* on the copper brown, light green, and mature leaves. **(C)** Die-back symptoms caused by *C. australisinense* on rubber tree leaves. **(E,F)** Anthracnose symptoms caused by *C. siamense* on mature rubber leaves.

In rubber tree plantations, *C. australisinense* mainly infects tender and immature leaves (phenophase: copper brown and light green leaves), as revealed initially by many brown conical or raised spots. As the disease progresses, the infected leaves become visibly wrinkled and twisted, eventually falling off the petiole (Figures 1A–C). On a mature leaf, the symptoms appear only as conical or raised spots, latterly perforated (Figure 1D). By contrast, the anthracnose spots caused by *C. siamense* infection appear as concentric rings that occur generally along the leaf margins, and occasionally in the middle of a mature leaf (Saha et al., 2002). These lesions are large, and may coalesce to form discernable larger-sized spots. The central portion of this spot is light brown and papery. Another noteworthy leaf spot symptom associated with *C. siamense* infection is the almost circular papery lesions having a dark brown center surrounded by a yellow halo (Saha et al., 2002; Figures 1E,F).

### Genome Sequencing and Assembly and Gene Prediction

The genome assembly size of *C. australisinense* GX1655 is distinctly different from *C. siamense*. BUSCO v3.0 was used to provide an estimate of assembly completeness. According to this analysis, the assembly of the *C. australisinense* genome covered 95.5% of the total gene space while that of *C. siamense* covered 99.3% of it (Table 1).

**TABLE 1:** Assembly and gene prediction information of *Colletotrichum* spp. genomes.

	***C. siamense***	***C. australisinense***
Assembly size (Mb)	58.4	55.3
Max contig (Mb)	7.62	10.04
N50 (Mb)	4.58	5.69
BUSCO complete	99.3%	95.5%
BUSCO partial	99.0%	95.5%
Repeat elements	1.85%	5.65%
Number of predicted genes	15493	15189
Average gene length (bp)	1612.97	1586.30
Average exons number per gene	2.90	3.17
Average introns number per gene	1.90	2.17

For the *C. australisinense* GX1655 genome, a total of 15,189 protein-coding genes were predicted, of which 26.9% (4095), 46.8% (7114), 58.4% (8870), 0.84% (127), and 98.2% (14,915) of the putative proteins could be, respectively, annotated based on COG, GO, Swiss-Prot, KEGGs, and NR, respectively. In the BUSCO analysis, 95.5% of the fungal core genes had “complete” hits and 95.2% had “complete and single-copy” hits, and ca. 5.65% of the assembly consisted of repeat sequences. From the *C. siamense* HBCG01 genome a total of 15,493 protein-coding genes were predicted—slightly more than obtained for the *C. australisinense* GX1655—of which 29.1% (4,519), 50.4% (7,812), 62.4% (9,672), 2.76% (427), and 99.0% (15,438) could be annotated based on COG, GO, Swiss-Prot, KEGGs and NR, respectively. According to BUSCO, 98.3 and 96.9% of this species fungal core genes had hits of “complete” and “complete and single-copy,” respectively, with repeat sequences amounting to ca. 1.85% of the assembly. For both species, the thoroughness of their genes’ annotation was demonstrated by the high percent values (Table 1). In the categorization of all genes based on GO, *C. siamense* HBCG01 in general has more genes in cellular component, molecular function, biological process. *C. australisinense* GX1655 has only higher abundance of genes involved in signal transducer activity and molecular transducer activity (molecular function), membrane-enclosed lumen (cellular component), biological regulation and cellular component organization or biogenesis (biological process) (Supplementary Figure 1).

### Phylogenetic Analyses

**FIGURE 2:**
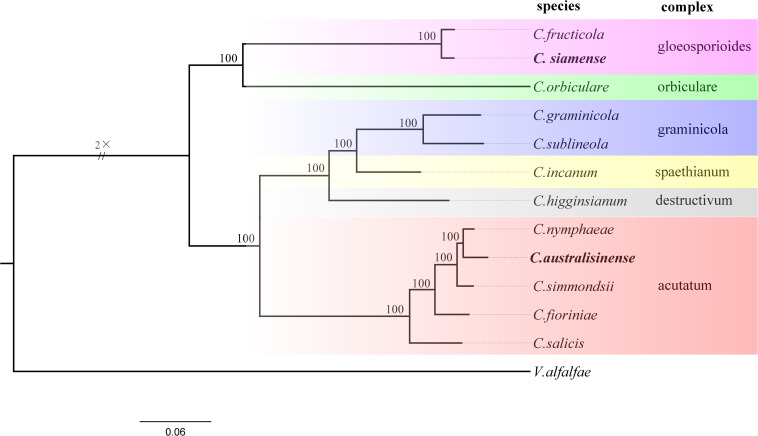
Phylogenetic tree based on single-copy orthologous genes of different *Colletotrichum* species, with *Verticillium alfalfae* as the outgroup to root the tree. All branches have 100% bootstrap support values. Two *Colletotrichum* species in this study are in bold.

In orthogroups analysis, we found 13,018 orthogroups in the *C. australisinense*, and 12,930 orthogroups in the *C. siamense*. A total of 4,782 single-copy orthologs were found in all species used in this study. 42 orthogroups had no homologs in any other species used in this study, and hence represented the species-specific orthogroups that are unique to *C. australisinense*. However, 5 orthogroups are species-specific to *C. siamense*. To understand the evolutionary relationships among species within the genus *Colletotrichum*, a phylogenomic tree was constructed based on the concatenated alignment of 4,782 single-copy orthologs obtained from the genomes of 12 *Colletotrichum* species. The inferred phylogeny showed that *C. australisinense* was most closely related to *C. nymphaeae*. *C. siamense* clustered with *C. fructicola*. The CAsc and CGsc appear to be evolutionary very distant (Figure 2).

### Secreted CAZymes

**FIGURE 3:**
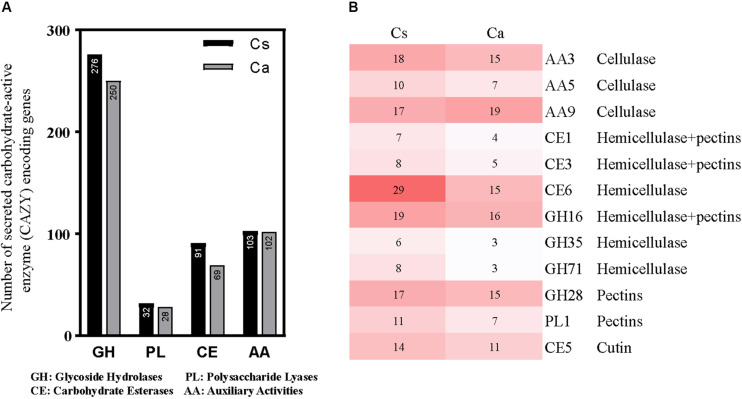
Analysis of different CAZyme families in *Colletotrichum siamense* and *C. australisinense*. **(A)** Total number and category of CAZymes in the predicted secretome, GH, glycoside hydrolases; PL, polysaccharide lyases; CE, carbohydrate esterases; AA, auxiliary activities. **(B)** Comparative analysis of important fungal and plant cell wall degrading CAZyme families in the genomes of the two studied fungal species. Cs, *C. siamense*; Ca, *C. australisinense.*

The plant cell wall primarily consists of three polysaccharide types (cellulose, hemicelluloses and pectins). Carbohydrate-metabolizing enzymes are vital for the degradation and utilization of these polysaccharides during host colonization by pathogenic fungi. In all, 451 genes of *C. australisinense* GX1655 associated with 88 CAZyme families were predicted to have secreted proteins; however, *C. siamense* HBCG01 harbored a larger repertoire of CAZymes in which 507 genes associated with 92 CAZyme families were predicted to have secreted proteins. The key classes of CAZymes, such as auxiliary activities (AA), carbohydrate esterases (CE), glycoside hydrolases (GH), and polysaccharide lyases (PL), were compared between species (Figure 3A). Evidently, *C. siamense* HBCG01 had higher copy numbers of these families, especially of CE and GH. Among the most amplified gene families were those involved in pectin degradation, such as PL1, GH28, and GH78, as well as those participating in the degradation of hemicelluloses and cellulose, such as CE1, CE3, CE6, GH16, GH35, GH71, and GH92 (Figure 3B). Hence, *C. siamense* evolution underwent a strong amplification of plant cell wall-degrading enzymes (PCWDEs). Auxiliary activities (AA) is another pertinent class of genes not belonging to CAZymes but instead linked to both biomass degradation and microbe-plant interaction (e.g., involved in breaking down lignin; Levasseur et al., 2013; Baroncelli et al., 2016). We found the total number of AA genes to be roughly similar in both species.

### Cytochrome P450s

**FIGURE 4:**
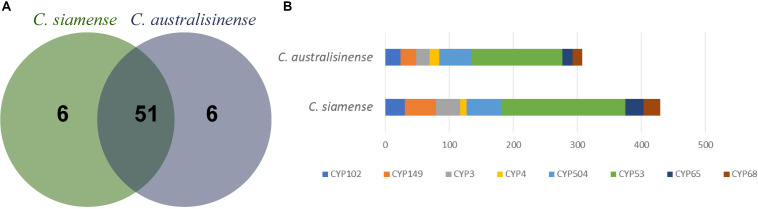
Number of cytochrome P450 monooxygenases gene families **(A)** and the number of major P450 families **(B)** in the two *Colletotrichum* species.

Cytochromes P450 (CYPs) are proteins of the superfamily containing heme as its cofactor, otherwise known as hemoproteins (Gonzalez and Gelboin, 1992). A total of 674 and 817 genes were predicted, respectively in *C. australisinense* and *C. siamense*, for which both numbers were associated with 57 families of CYPs (Figure 4A), which pointed to a significant higher abundance in P450 gene families of *C. siamense.* The family that expanded most was CYP53. Research on fungal P450s has revealed that the P450 family CYP53 can serve as a novel alternative anti-fungal drug target (Jawallapersand et al., 2014), and the CYP53 gene was earlier shown to be essential for fungal species survival (Fraser et al., 2002). The CYP149, CYP68, and CYP65 families corresponding to cytochrome P450s related to SM biosynthesis (Jimbo et al., 2000; Liang et al., 2018) also have obvious higher abundance (Figure 4B).

### SM Synthesis Capacity

**FIGURE 5:**
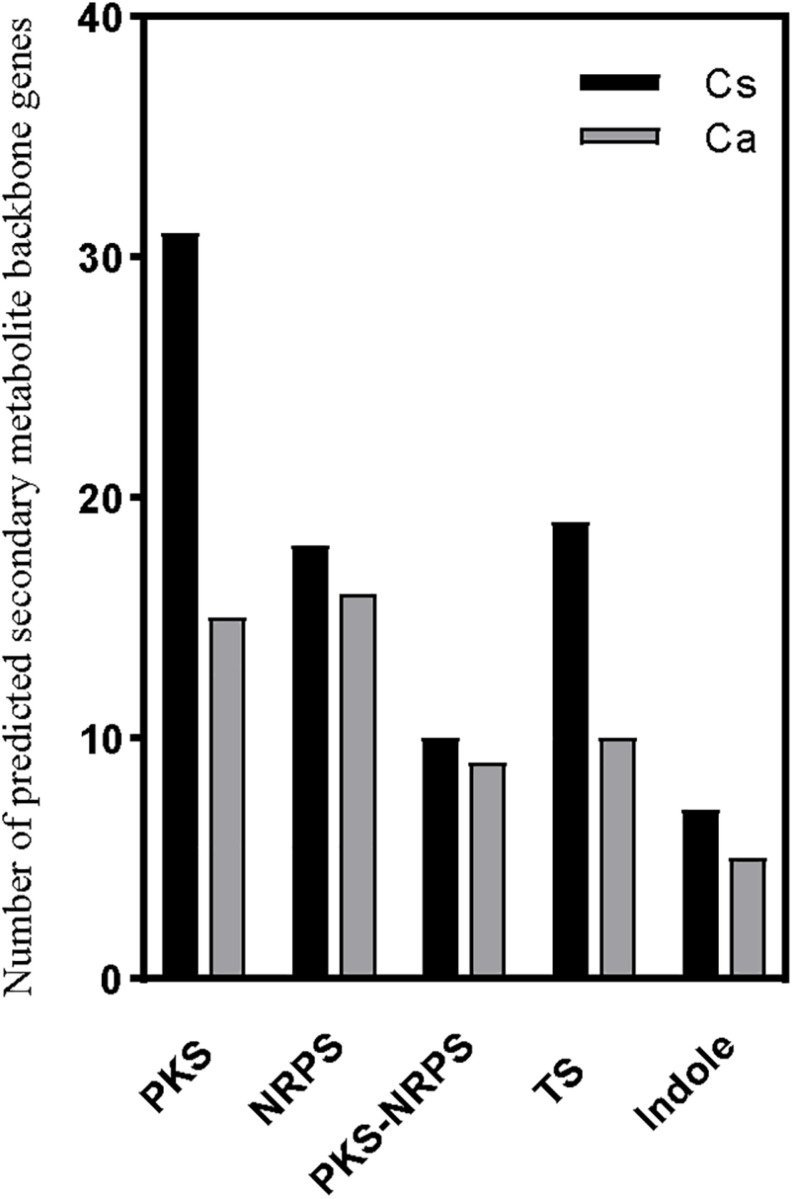
Number of secondary metabolite backbone genes predicted by antiSMASH in the two species of *Colletotrichum*. PKS, polyketide synthase; NRPS, non-ribosomal peptide synthetase; PKS-NRPS, PKS-NRPS hybrid containing at least one PKS and one NRPS domain; TS, terpene synthase. Cs, *C. siamense*; Ca, *C. australisinense.*

The fungal genus *Colletotrichum* produces an enormous array of SM that could figure prominently in its pathogenesis of plants. The biosynthesis of SM requires precursor genes, most of which are located inside fungal gene clusters. It is imperative to identify the genes involved in the codification of these enzymes, because these genes are often involved in different mechanisms of pathogenicity and/or virulence (Boettger and Hertweck, 2013; Moraga et al., 2018). Here, by using antiSMASH, 85 SM gene clusters in the *C. siamense* genome were predicted: 31 type 1 polyketide synthases (t1PKS) and 18 NRPS genes, 7 t1PKS-NRPS hybrid genes, 19 terpene (TS) and 7 indole genes, and one each of t1PKS- type 3 polyketide synthases (t3PKS), NRPS-TS, and t1PKS-indole hybrid genes. Yet there were fewer SM gene clusters in the *C. australisinense* genome, whose 55 clusters comprised 15 t1PKS and 16 NRPS genes, 7 t1PKS-NRPS hybrid genes, 10 TS and 5 indole genes, and one each of NRPS-betalactone and NRPS-TS hybrid genes. The t1PKSs commonly responsible for the biosynthesis of macrolides were the most abundant clusters detected in the *C. siamense* genome (Figure 5). Some of these genes showed homology to known clusters in other fungi: eight gene clusters of *C. siamense* showed 100% similarity with alternapyrone (*Alternaria solani*), dimethylcoprogen (*Alternaria solani*), chrysogine (*Fusarium graminearum*), clavaric acid (*Hypholoma sublateritium*), fusarin (*Fusarium verticillioides*), phomopsins (*Phomopsis leptostromiformis*), gibberellin, and 1,3,6,8-tetrahydroxynaphthalene (*Nodulisporium* sp. ATCC74245) biosynthetic gene clusters. Of these clusters, in *C. australisinense* genome only alternapyrone and 1,3,6,8-tetrahydroxynaphthalene biosynthetic gene clusters were found, which showed 100% similarity. Furthermore, two species-specific gene clusters of *C. australisinense* showed 100% similarity with the pyranonigrin E (*Aspergillus niger* ATCC 1015) and deoxysambucinol/sambucinol/roridin E (*Fusarium sambucinum*) biosynthetic gene clusters.

**FIGURE 6:**
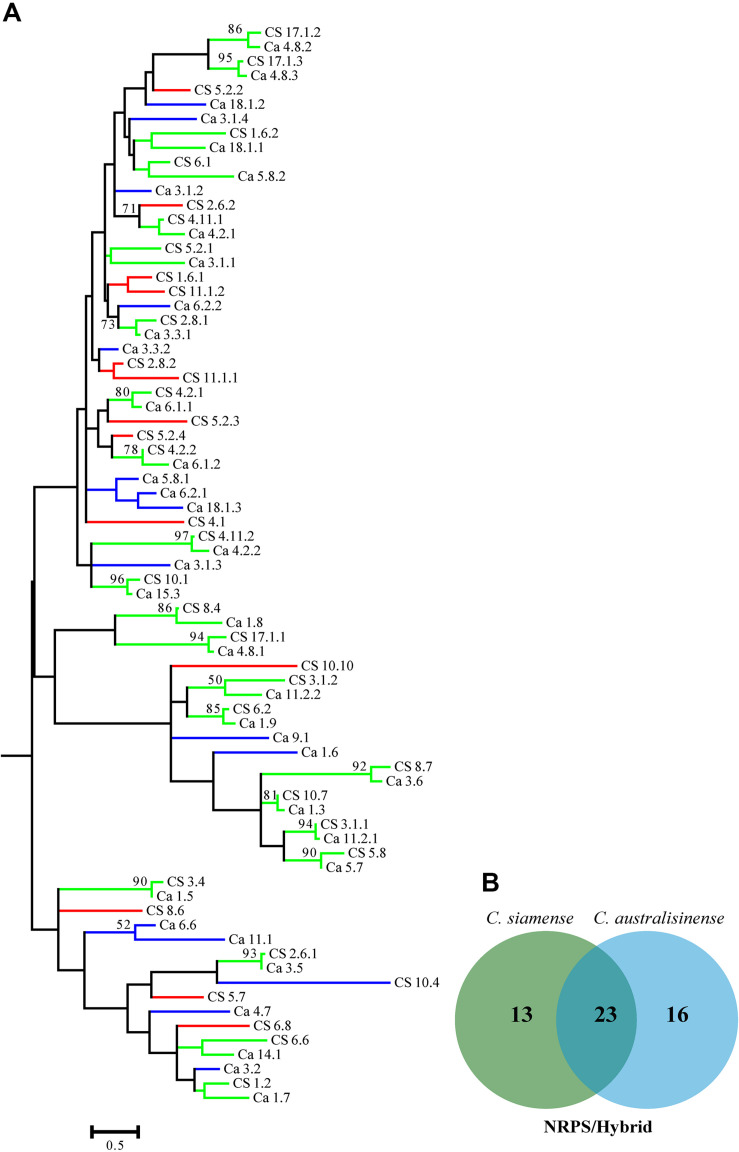
**(A)** Phylogenetic tree of the adenylation domain (A domain) amino acid sequences of putative NRPS and PKS-NRPS hybrids. Sequences were aligned using MEGA 6.0, and the phylogenies inferred by maximum-likelihood (as calculated by the LG + G Model) in MEGA 6.0. The numbers on branch nodes indicate support values above 50%. Red branches show the A domain of *Colletotrichum siamense* only; blue branches show the A domain of *C. australisinense* only; green branches the A domain shared by both species. **(B)** Venn diagram summarizing the numbers of conserved and non-conserved sequences between the two fungal species.

**FIGURE 7:**
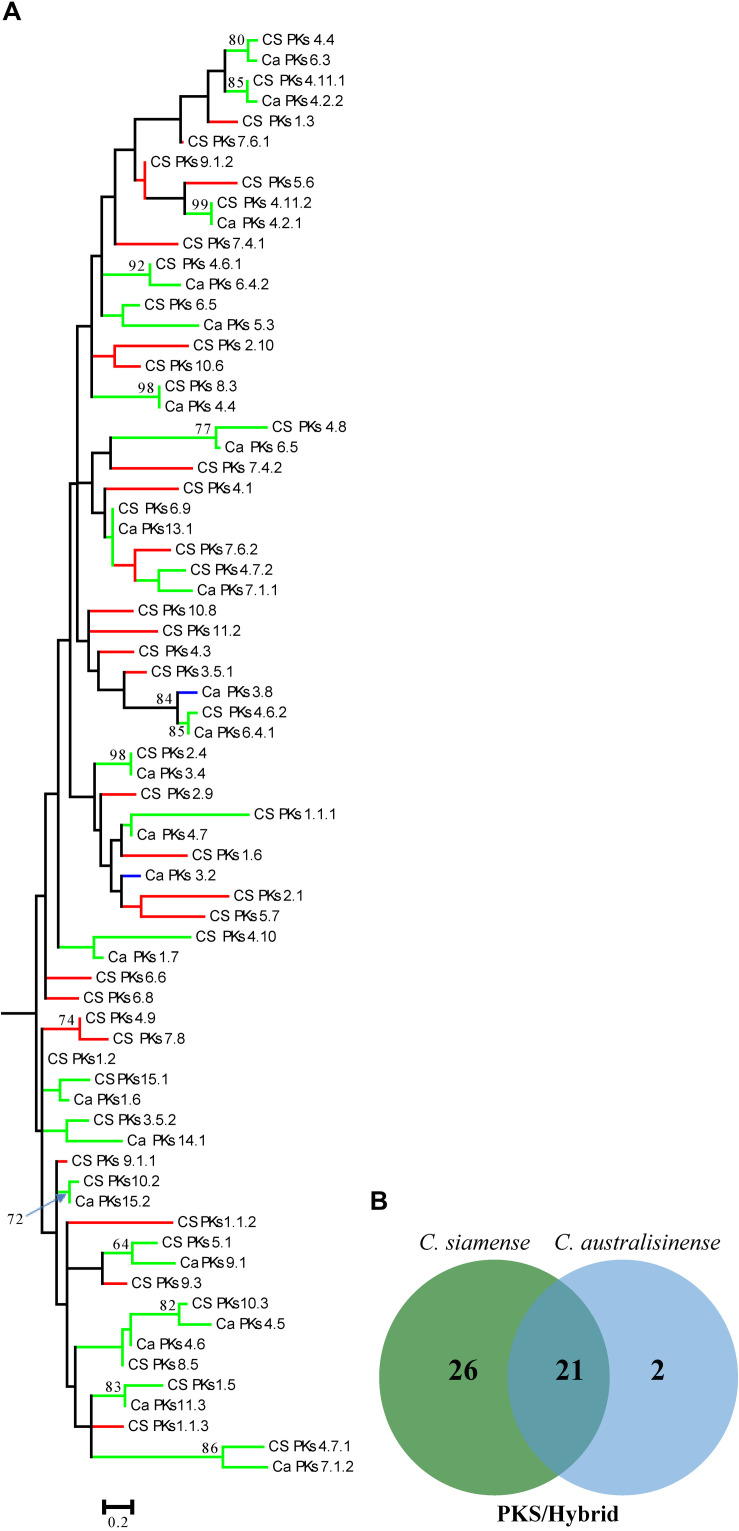
**(A)** Phylogenetic tree of the keto-synthase (KS) domain amino acid sequences of putative PKSs and PKS-NRPS hybrids. Sequences were aligned using MEGA 6.0, and phylogenies inferred by maximum-likelihood (as calculated by the LG + G Model) in MEGA 6.0. The numbers on branch nodes indicate support values above 50%. Red branches show the KS of *Colletotrichum siamense* only; blue branches show the KS of *C. australisinense* only; green branches are the KS shared between both species. **(B)** Venn diagram summarizing the numbers of conserved and non-conserved sequences between the two fungal species.

We also investigated the relationships among the putative SM-associated proteins using the adenylation domain (A domain) of NRPS proteins and the keto-synthase (KS) of PKS in the two *Colletotrichum* spp. This phylogenetic analysis revealed a rich diversity, with relatively few NRPS-associated protein ortholog families conserved across both species (Figures 6A,B). Nonetheless, 11 PKS gene clusters—but none that included the PKS-NRPS hybrid—were apparently shared between *C. siamense* and *C. australisinense*. Interestingly, *C. siamense* HBCG01 had a larger repertoire of keto-synthase (KS) in these PKS clusters (Figures 7A,B).

### Necrosis and Ethylene-Inducing Peptide 1 (Nep1)-Like Proteins (NLPs)

**FIGURE 8:**
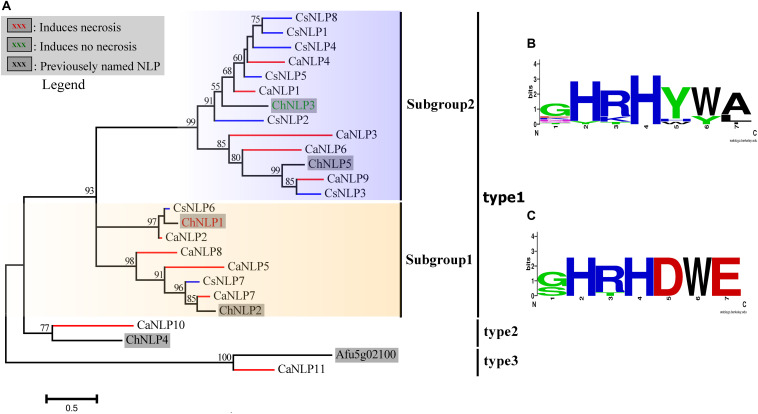
**(A)** Phylogenetic reconstruction of secreted necrosis- and ethylene-inducing peptide 1 (Nep1)-like proteins (NLPs). Blue branches show the NLPs of *Colletotrichum siamense*; red branches show the NLPs of *C. australisinense*. **(B)** Aligned Weblogo of the conserved motif GHRHDWE sequence of subgroup-2. **(C)** Aligned Weblogo of the conserved motif GHRHDWE sequence of subgroup-1. ChNLP1, ChNLP2, ChNLP3, ChNLP4, ChNLP5 (Kleemann et al., 2012) and Afu5g02100 (Upadhyay et al., 2012) were used for reference sequence of different type NLP (Oome and Van den Ackerveken, 2014).

Nep1-like proteins (NLP) are perhaps best known for their cytotoxic activity in dicot plants (Oome and Van den Ackerveken, 2014). These proteins function as effectors that boost pathogen virulence during the host colonization process by disintegrating the plasma membrane of plant cells. NLP are taxonomically widespread among microbes having very different lifestyles (Oome and Van den Ackerveken, 2014). Here, NLPs were identified through a search for the conserved motif GHRHDWE in the predicted proteins. Eleven proteins were predicted in *C. australisinense*, and likewise eight in *C. siamense*, which belonged to the NEP1-like protein (NLP) family (Oome and Van den Ackerveken, 2014). Two NLPs of *C. siamense*, lacking their N-terminal sequence, were not included in this study, perhaps because of incomplete genome sequencing or due to being pseudogenes. Clear differences in both the type and number of NLPs between the two *Colletotrichum* species were obtained. Among the predicted NLPs, nine of *C. australisinense* were identified as type-1, plus one each of type-2 and type-3. In stark contrast, all NLPs for *C. siamense* belonged to type-1. According to the substitutions in the conserved GHRHDWE heptapeptide motif, diversification of type-1 NLP is discernable, in that it could be divided into two subgroups (i.e., subgroup-1 and subgroup-2). The subgroup-1 NLP contained a near fully conserved motif, whose members are confirmed participants in the induction of necrotrophy. However, the aspartic acid and glutamic acid residues (underlined) of the conserved heptapeptide motif (GHRHDWE) are not conserved in subgroup-2 NLP, whose members lack necrosis-inducing activity (Figure 8; Kleemann et al., 2012; Oome and Van den Ackerveken, 2014).

## Discussion

In our study, the whole-genome sequencing, assembly, and functional annotation of *C. australisinense* and *C. siamense* characterized by distinct lifestyles on rubber tree were carried out. Pathogenicity-related genes were classified and compared, and marked duplication/reduction events were also identified. *Colletotrichum* is a large genus of *Ascomycete* fungi, containing many species with different life-histories capable of causing anthracnose or blight to a wide range of host plants (Jayawardena et al., 2016). The majority of *Colletotrichum* spp. are known for their hemibiotrophic lifestyle, which includes two successive stages of infection, biotrophy followed by necrotrophy. The particular life history of species in this genus depends on multiple factors, including the host identity, type of host tissue infected, and local environmental conditions (Perfect et al., 1999; De Silva et al., 2017). Although *C. australisinense* and *C. siamense* form the dominant populations causing *Colletotrichum* leaf disease in rubber trees, they have a different life history. *C. australisinense* has only been found on the rubber tree. By contrast, the *C. siamense* has a wide host range, and can colonize many host plant species, such as those in the genera *Carica*, *Coffea*, *Mangifera*, *Annona*, *Musa*, and *Camellia* (Udayanga et al., 2013; Liu et al., 2015). Genus-wide comparative genome analyses indicate that *Colletotrichum* species have tailored profiles for some enzymes according to their specific lifestyle, including CAZymes, SM synthetases, and peptidases (Gan et al., 2016; Buiate et al., 2017; De Silva et al., 2017; Liang et al., 2018). In the pathogenic process, these genes are expressed dynamically to fulfill stage-specific pathogenic functions (O’Connell et al., 2012; Gan et al., 2013; Rao and Nandineni, 2017). Effectors and SM enzymes are induced before penetration and during biotrophy, whereas most degradative enzyme and transporters are upregulated later, at the necrotrophic stage of growth (O’Connell et al., 2012). Hemibiotrophy in *C. orbiculare* is characterized by distinct stage-specific gene expression profiles of expanded classes of potential pathogenicity genes (Gan et al., 2013). The comparative genomic research has ever yielded key insights into the *Colletotrichum* fungi’s evolution (Liang et al., 2018). The gene family expansions and contractions are thought to be critical for shaping the host specificity of members of this genus (Gan et al., 2016; Baroncelli et al., 2016).

When compared with *C. australisinense*, our results suggests that the major polysaccharide degrading enzyme (CAZyme) families underwent a species-specific increase of pectin degrading CAZymes (GH28, GH78, PL1), cutinases (CE5), and hemicelluloses and cellulose degrading CAZymes (CE1, CE3, CE6, GH16, GH35, GH71, GH92) in *C. siamense*, the species that causes anthracnose symptoms in a wide variety of hosts. Thus, *C. siamense* has a stronger ability to degrade plant cell wall components, such as xyloglucan, xylan, pectin, and cellulose. This offensive trait may help explain why, of the two *Colletotrichum* spp. studied, it is *C. siamense* which mainly infects the mature leaves of rubber tree, causing large anthracnose lesions. In contrast, the *C. australisinense* infection of leaves only causes little raised spots that slowly expand.

P450s are diverse, heme-thiolate proteins, which enable primary and SM synthesis, fungal pathogenicity, and detoxification of plant-derived antimicrobial compounds. In this respect, there seems to be little difference between *C. siamense* and *C. australisinense*, both of which contain nearly the same P450 categories. However, the gene number of particular P450 families has strongly expanded in *C. siamense*, especially that of CYP65, while CYP68 was found closely associated with the SM synthesis, and CYP102, CYP149, and CYP3 with toxic substance metabolism. These results correspond well with the number of SM gene clusters predicted in two species, which may explain how these two pathogens can detoxify phytoalexins (Soby et al., 1996).

The SMs produced by fungal phytopathogens are typically associated with their respective pathogenicity and host range (Rao and Nandineni, 2017). When applied to host leaves, these phytotoxic metabolites will induce symptoms similar to those of anthracnose caused by *Colletotrichum* species, and they have been shown to play a significant role in pathogenesis and infection mechanisms (García-Pajón and Collado, 2003). The comparative analysis of the SM gene clusters, as predicted through antiSMASH, revealed considerably more of such clusters in *C. siamense*, especially PKS (31 vs. 15) and TS (19 vs. 10) are twofold higher than in *C. australisinense*. Although most of the KS genes in the predicted *C. australisinense* PKS clusters were shared with *C. siamense*, the latter did harbor many specific KS genes that could contribute to its genetic potential to generate diverse SMs. It has been appreciated for some time now that PKSs are important in facilitating host penetration by *Colletotrichum* species (Takano et al., 1995, 1997).

Nep1-like proteins (NLPs) induce necrosis and ethylene production when they infiltrate the extracellular space in leaves of dicot plant species (but not monocots) (Oome and Van den Ackerveken, 2014). The NLPs are taxonomically widespread among microbes having very different lifestyles. Based on their phylogenetic analysis of NLP sequences, Oome and Van den Ackerveken (2014) defined four groups belonging to three phylogenetic types. A great number of newly identified NLP sequences, from a broad range of diverse organisms, have been studied to date, providing much valuable insight into the evolution and functions of these remarkable proteins (de Oliveira et al., 2012; Kleemann et al., 2012; Zhou et al., 2012; Oome and Van den Ackerveken, 2014; Chen et al., 2018). Importantly, each NLP type differs in its ability to induce necrosis. It has been suggested that an NLP acts in a manner extracellular to the plant cell to confer its cytotoxic activity, which may be achieved via different mechanisms (Kleemann et al., 2012; Oome and Van den Ackerveken, 2014). In this study, we found that the number and categories of NLPs in *C. australisinense* exceeded those of *C. siamense*: In the previous work, it was confirmed that ChNLP1 and ChNLP2 of *C. higginsianum* were upregulated during the transition from biotrophy to necrotrophy, the former having been shown to induce necrosis in plants. Most of the NLPs in *C. siamense* belong to subgroup II, including ChNLP3 and ChNLP5, which are unable to induce necrosis in infected host plants. Since the genes encoding non-cytotoxic NLP are expressed during the biotrophic and early stages of infection by these (hemi)biotrophic pathogens, this suggests they play a role in the penetration or establishment of infection (Kanneganti et al., 2006; Kleemann et al., 2012). Previous studies have revealed lineage-specific expansions of NLP families within the *C. acutatum* species complex. It may well be that a lineage specific evolution is unique to members of the *C. acutatum* species complex (Baroncelli et al., 2016; Liang et al., 2018).

The remarkable repertoire of NLPs found in the *C. acutatum* species complex might be linked to their potent ability to infect multiple host species. Some of these proteins may function as necrosis-inducing proteins, whereas other NLPs may contribute to overcoming early plant defense responses (Baroncelli et al., 2016). We suggest that *C. australisinense*—which belongs to the *C. acutatum* species complex—has a stronger ability to induce necrosis than does *C. siamense* of the *C. gloeosporioides* species complex. For species within the *acutatum* complex, infection strategies are usually based on intracellular hemibiotrophy in addition to subcuticular, intramural necrotrophy (De Silva et al., 2017). Nonetheless, infection by *C. gloeosporioides* was reportedly hemibiotrophic, in that both intracellular hemibiotrophic and intramural necrotrophic infections can occur (Kim et al., 2004; O’Connell et al., 2012; Moraes et al., 2013; De Silva et al., 2017). For the intracellular hemibiotrophic strategy, transitioning from one state to another can vary depending on the host plant identity and ontogenic stage and the particular infecting *Colletotrichum* species, of which some engage in rather unique infection behaviors. Species in the *acutatum* complex commonly begin their life cycle with a short biotrophic phase before switching to a necrotrophic stage (De Silva et al., 2017). These developmental aspects may help to explain why *C. australisinense* mainly infects tender and immature leaves of rubber tree, leading to rapidly wrinkled lesions.

## Data Availability Statement

The datasets generated for this study can be found in the GenBank, accession nos. PRJNA597657 and PRJNA597926.

## Author Contributions

XL conducted the experiments and analyzed the results. BL, YY, JC, TS, and XZ collected *Colletotrichum* isolates and performed the experiments. GH revised and approved the final version of the manuscript. All authors reviewed the manuscript.

## Conflict of Interest

The authors declare that the research was conducted in the absence of any commercial or financial relationships that could be construed as a potential conflict of interest.

## Footnotes

1 https://github.com/lbcb-sci/graphmap2

2 http://tlife.fudan.edu.cn/tlife/ltr_finder/

3 www.cazy.org

4 http://cyped.biocatnet.de/

5 http://www.cbs.dtu.dk/services/TargetP/

6 http://mafft.cbrc.jp/alignment/server
