# Supplementary material for: Pathogenic Adaptations Revealed by Comparative Genome Analyses of Two Colletotrichum spp., the Causal Agent of Anthracnose in Rubber Tree
Source: Front Microbiol. 2020 Jul 21;11:1484. doi: 10.3389/fmicb.2020.01484 (PMC7385191; doi:10.3389/fmicb.2020.01484)
Supplement: Supplementary file 1 [file Data_Sheet_1.docx]

Supplementary Material

## Supplementary Figures


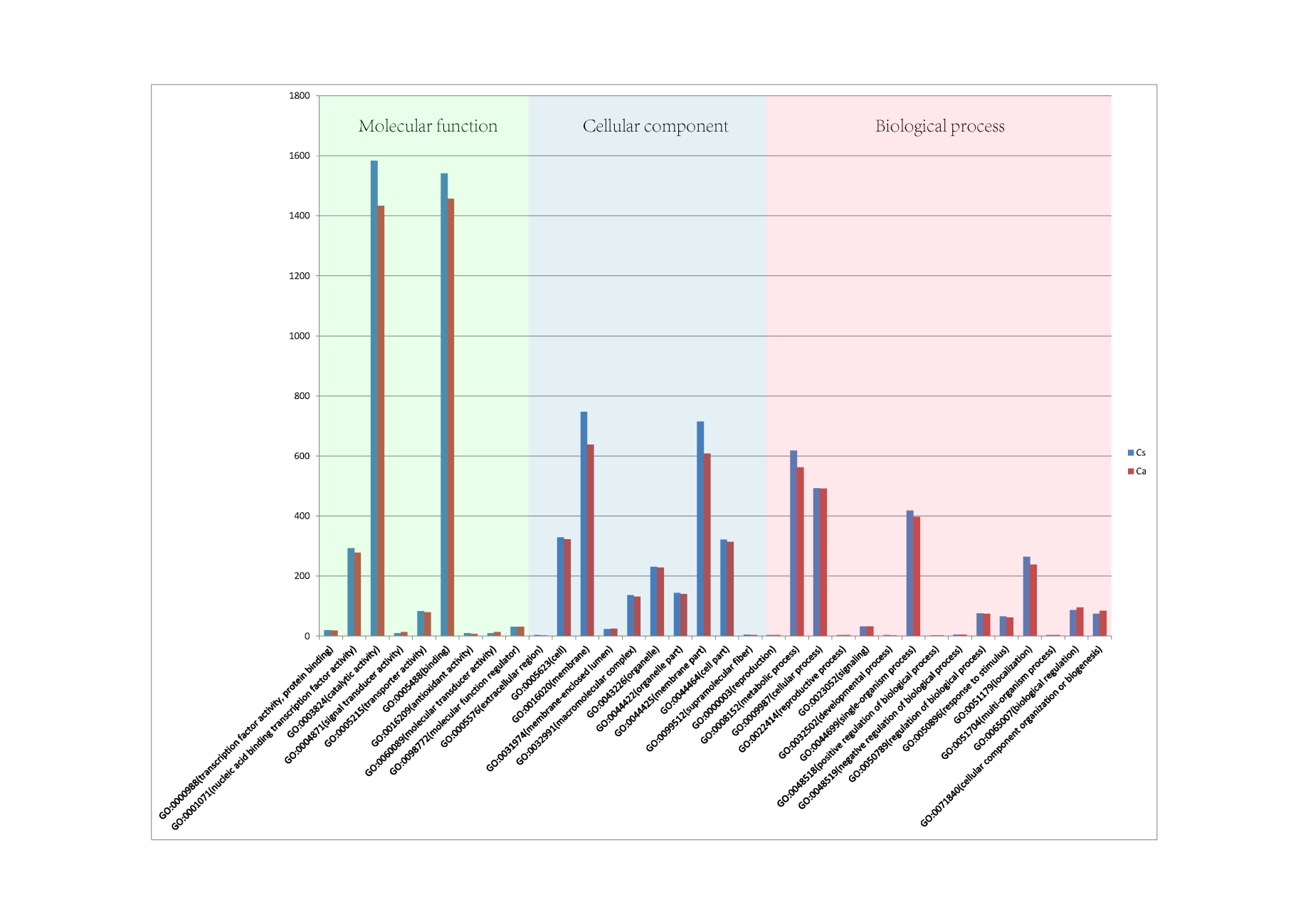


**Supplementary Figure 1**. **The categorization of all genes based on GO (Gene ontology - cellular component, molecular function, biological process) of *C. siamense* and *C. australisinense*. Cs: *C. siamense*; Ca: *C. australisinense*.**
